# The impact of presentation modes on mental rotation processing: a comparative analysis of eye movements and performance

**DOI:** 10.1038/s41598-024-60370-6

**Published:** 2024-05-29

**Authors:** Philipp Stark, Efe Bozkir, Weronika Sójka, Markus Huff, Enkelejda Kasneci, Richard Göllner

**Affiliations:** 1 Hector Research Institute of Education Sciences and Psychology, University of Tübingen, Europastraße 6, 72072 Tübingen, Germany; 2 Department of Psychology, University of Tübingen, Schleichstraße 4, 72076 Tübingen, Germany; 3 Human-Computer Interaction, University of Tübingen, Sand 14, 72076 Tübingen, Germany; 4 Human-Centered Technologies for Learning, Technical University of Munich, Arcisstraße 21, 80333 Munich, Germany; 5 Perception and Action Lab, Leibniz-Institut für Wissensmedien, Schleichstraße 6, 72076 Tübingen, Germany; 6 Institute of Educational Science, Faculty of Human Sciences, University of Regensburg, Universitätsstraße 31, 93053 Regensburg, Germany

**Keywords:** Human behaviour, Psychology

## Abstract

Mental rotation is the ability to rotate mental representations of objects in space. Shepard and Metzler’s shape-matching tasks, frequently used to test mental rotation, involve presenting pictorial representations of 3D objects. This stimulus material has raised questions regarding the ecological validity of the test for mental rotation with actual visual 3D objects. To systematically investigate differences in mental rotation with pictorial and visual stimuli, we compared data of $N=54$ university students from a virtual reality experiment. Comparing both conditions within subjects, we found higher accuracy and faster reaction times for 3D visual figures. We expected eye tracking to reveal differences in participants’ stimulus processing and mental rotation strategies induced by the visual differences. We statistically compared fixations (locations), saccades (directions), pupil changes, and head movements. Supplementary Shapley values of a Gradient Boosting Decision Tree algorithm were analyzed, which correctly classified the two conditions using eye and head movements. The results indicated that with visual 3D figures, the encoding of spatial information was less demanding, and participants may have used egocentric transformations and perspective changes. Moreover, participants showed eye movements associated with more holistic processing for visual 3D figures and more piecemeal processing for pictorial 2D figures.

## Introduction

Mental rotation, the ability to rotate mental representations of objects in space, is a core ability for spatial thinking and spatial reasoning^1,2^. Mental rotation is required for everyday skills, like map reading or navigating, and is an important prerequisite for individuals’ learning^3^. Higher mental rotation performance is associated with higher fluid intelligence and better mathematical thinking^4^. It has been found to be beneficial for students’ learning in mathematics domains such as geometry and algebra^5^. Thus, mental rotation ability acts as a gatekeeper for entering STEM-related fields in higher education^6^.

A standardized test by Shepard and Metzler^7^ for measuring humans’ mental rotation performance displays two-dimensional (2D) images of two unfamiliar three-dimensional (3D) figures. For these pictorial stimuli, participants are instructed to determine whether the two figures are identical. For this, the two figures are depicted from different perspectives by independently rotating one of them along its axis^7,8^. Individuals’ performance in mental rotation is reflected by the number of correct answers and task-solving speed (reaction time)^9,10^. Since its initial development, this experiment has been replicated many times^10–13^. The test by Shepard and Metzler is one of the most frequently used tests to examine mental rotation. It laid the foundation for understanding spatial cognition^14–17^ and continues to be referenced in contemporary research^10,18,19^. Replicating this classic experiment allows researchers to build on a well-established foundation and examine enduring principles of mental rotation.

However, its ecological validity to assess real-life mental rotation has been questioned^20,21^. Developments in the field of virtual simulations enable experiments to be conducted with increased ecological validity yet still under controlled and standardized conditions^22^. In particular, virtual realities (VR) have become powerful tools in psychological research^23,24^. VR allows for the creation of environments with 3D spatial relations that can be explored and manipulated by users and are experienced in an immersive way^25^. This allows for the presentation of visual 3D figures, rendered as 3D objects in the environment, and introduces visual and perceptual differences to pictorial (2D) stimuli.

The pictorial stimuli of the conventional mental rotation test are orthographic, parallel representations of 3D figures on a planar surface (as images). This pictorial representation lacks two sources of depth information present in visual (3D) figures when placed in a VR environment with realistic spatial relations^26^. The first source of depth information is provided by stereoscopic vision due to binocular disparity. The binocular disparity stems from the slight offset between the two displays projected onto the two eyes in the head-mounted display (HMD), enabling stereopsis and depth perception^27^. This depth cue is particularly relevant for 3D vision, where it contributes to participants’ ability to perceive depth and spatial relationships between objects. The second source of depth information is introduced by motion parallax^26,28^. Motion parallax, also known as structure-from-motion, emerges as a consequence of real-time head tracking and rendering based on the observer’s position within the virtual space. This dynamic depth cue allows users to perceive the 3D structure of objects by moving their heads. As they move relative to the 3D object, the representation of the object is updated and provides different views to identify the object. Furthermore, shadows provide additional depth information. They occur when physical objects interact with light sources in a VR environment. Shadows contribute to the perception of object volume and spatial relationships in visual figures. Presenting mental rotation stimuli in VR provides the most comprehensive visual information. In contrast, rear-projection systems offer solely pictorial information^29^, and stereoscopic glasses introduce binocular disparity^30^, leaving motion parallax as the final piece of the puzzle added by VR^31^.

This additional visual information is expected to affect participants’ stimulus processing and mental rotation strategy when solving items with visual stimuli in comparison to pictorial representations. A series of processing steps when solving mental rotation tasks have been identified^32,33^: (1) encoding and searching, which combines the perceptual encoding of the stimulus and the identification of the stimulus and its orientation; (2) transformation and comparison, which includes the actual process of mentally rotating objects; (3) judgment and response, which combines the confirmation of a match or mismatch between the stimuli and the response behavior.

One would expect the visual modes of presentation to introduce differences in the processing steps. During encoding and searching with pictorial figures, a model of the 3D object structure must be recovered from a planar 2D representation^34^. This reconstruction process has been found to be a demanding task^35^ and should not be necessary with visual figures. One would also expect the identification of the stimulus and its orientation to be more demanding with pictorial figures. A displayed image remains static regardless of the observer’s location; therefore, participants have to make assumptions about occluded or ambiguous parts of the figure. For pictorial figures, the additional head movement might even produce perceptual distortions described by the differential rotation effect^36^, in which the size and shape of images are perceived inappropriately when the observer is not in the center of the projection^37^. In contrast, binocular disparity and motion parallax would constantly update the visual 3D figures based on the participants’ relative location to the object. Test takers can explore the visual figures and gather additional information from different perspectives, which should help them to identify the figures and their orientation more easily.

In the second step of transformation and comparison, mental rotation involves manipulating and rotating mental representations of geometric figures in the mind. Exploiting motion parallax with visual 3D figures could reduce the need for extensive mental transformations. For example, participants could reduce the rotation angle between the figures through lateral head movement. The rotation angle is the degree to which the figures are rotated against each other. This may make the comparison process more intuitive and less cognitively demanding. Motion parallax due to head movement could also lead to a shift from the object-based transformation of the stimuli to an egocentric transformation^38^. In object-based transformations, the observer’s position remains fixed while the object is mentally rotated. An egocentric transformation involves a change of perspective, rotating one’s body to change the viewpoint and orientation. It has been found that egocentric transformations, as a form of self-motion, are more intuitive and result in faster and more accurate mental rotation^39^.

Similar reaction times for mental and manual rotation suggest that participants mentally align the figures to each other for comparison^40^. Two prominent alignment strategies have been described for mental rotation: piecemeal and holistic. The piecemeal strategy involves breaking down the object into segments and mentally rotating the pieces in congruence with the comparison object to assess their match. A holistic approach entails mentally rotating the entire object and encoding comprehensive spatial information about it^41,42^. In their original study, Shepard and Metzler viewed the linear relationship between rotation angle and reaction time as evidence against conceptual or propositional processing of visual information^7,43^. Later research, which investigated the process of rotation itself, revealed that both a holistic and a piecemeal approach were used to align the figures^16,42,44^. When processing visual figures, motion parallax allows for lateral head movements, which could be used to decrease the rotation angle between the figures by changing perspectives. The additional depth information due to binocular disparity could facilitate the comparison of spatial relationships between object features. These aspects might enable a more holistic processing of the figures.

Regarding judgment and response, participants are expected to perform better with visual 3D figures than with pictorial 2D figures. Lower cognitive demands during encoding might result in faster stimulus processing. The potential to apply an egocentric transformation and more holistic processing can be expected to lead to more efficient and more accurate responses with visual 3D figures.

The process of mental rotation is reflected in eye movements, which capture the visual encoding of spatial information^13,33^. Eye movement metrics can provide comprehensive information on stimulus processing and mental rotation strategies^13,41,42,45–47^. Basic experiments have shown that eye movements are controlled by cognitive processes, and consequently, it is possible to distinguish task-specific processes^48^. For example, different mental rotation strategies were identified and discriminated based on fixation patterns derived from eye-tracking data^16^. Fixation measures that incorporate spatial information are expected to reveal relevant information about stimulus processing. Different fixations on different segments of the figures have been associated with the first or second processing steps^33^. During the step of encoding and searching, the majority of fixations targeted one segment of one figure, whereas in the second step of transformation and comparison, fixations targeted all segments of both figures equally. This should lead to a higher fixation duration on singular segments in the first step and an equal fixation duration on all parts of the figure in the second step.

Saccadic movements between fixations, measured by saccade rate or saccade velocity, have also been utilized to investigate mental rotation with pictorial figures^33,46,49^. Directional saccadic movements containing spatial information can reveal temporal dependencies in stimulus processing^33^. For example, a backward saccade that guides the eye toward a previous location is called a regressive saccade^50^. We would expect that the regression towards a previous location could either be a need for information retrieval of figure information or a back-and-forth between congruent figure segments during the comparison step.

Regarding mental rotation strategies, information about the number of transitions between figures compared to the number of fixations within the figures has been applied to quantify the use of holistic vs. piecemeal strategies^42,46^. The ratio of the number of within-object fixations divided by the number of between-objects fixations has been shown to indicate holistic processing (ratio $\le 1$) or piecemeal processing (ratio $> 1$)^42,51^.

The pupil diameter provides information about the size of the pupil in both eyes and can be used to detect changes due to contraction and dilation. An increase in pupil diameter has been associated with higher cognitive load^52–54^, as the Locus Coeruleus (LC) controls pupil dilation and is engaged in memory retrieval^55,56^. Moreover, two different measures of pupil diameter behavior have been attributed to the phasic and tonic modes of LC activity^55^. Tonic mode activity is indicated by a larger overall pupil diameter and is associated with lower task utility and higher task difficulty. Phasic mode activity is indicated by larger pupil size variation during the task and is associated with task engagement and task exploitation^13,57^. While solving mental rotation tasks, a larger average pupil diameter over individual trials could indicate tonic activity, whereas a larger peak pupil diameter as a task-evoked pupillary response could indicate phasic activity^13,56^.

Recently available devices for analyzing eye movements in VR experiments include eye-tracking apparatuses. These devices record sensory data frame by frame to track visual and sensorimotor information in a standardized way during experiments^58^. The VR’s HMD additionally allows for tracking head movement. Changes in head movement serve as a valuable indicator of whether participants make use of motion parallax. A recently published study by Tang et al.^46^ analyzed eye movements during a mental rotation task in VR, but solely for visual 3D figures. The results of their VR experiment showed that the mental rotation test with visual 3D figures replicates the linear relationship between rotation angle and reaction time. Lochhead et al.^31^, on the other hand, investigated performance differences between pictorial and visual 3D figures presented in VR. Their results indicated that participants exhibited higher performance in the 3D condition compared to the 2D condition. However, they did not use eye tracking to capture participants’ visual processing of the stimuli to potentially explain presentation mode effects on performance.

Our study used a VR laboratory (see Fig. 2) to examine individuals’ mental rotation performance for pictorial 2D figures and visual 3D figures with the Shepard and Metzler test. We examined eye and head movements from $N = 54$ university student participants to determine differences in stimulus processing and mental rotation strategies when solving mental rotations with pictorial and visual stimuli. In both conditions, 28 stimuli pairs were shown, modeled after the original figures by Shepard and Metzler^7^. In the 3D condition, stimuli were rendered on a virtual table in front of the participants, allowing them to view the figures from different perspectives by moving their heads. In the 2D condition, the stimuli appeared on a virtual screen placed on the table at the same distance from the participants as in the 3D conditions. A series of 3D and 2D figures were presented, with the two conditions randomized block-wise within each student. For each task, participants’ performance in terms of the number of correct answers and reaction time as well as eye-movement features were recorded. The following hypotheses were formulated:

First, we expected participants’ performance in solving mental rotation tasks to be better with visual 3D figures than with pictorial 2D figures. Second, we expected the visual differences to evoke differences in stimulus processing and mental rotation strategies, which may indicate differences in performance between the two modes of presentation. To investigate this hypothesis, we analyzed how eye and head movements differed during task-solving in both conditions. To ensure that we could compare all stimulus pairs between the two conditions, no overall time limit was set for the experiment.

In addition to utilizing statistical analysis, we implemented a Gradient Boosting Decision Tree (GBDT)^59^ classification algorithm to identify the experimental condition based on eye and head movements. This machine learning approach surpassed traditional linear statistical methods, which are often limited to linear relationships between features and the target variable. Successfully predicting the experiment condition based on eye and head movement features would demonstrate the importance of these features for the distinguishing task.

Behavioral data, such as eye and head movements, are characterized by temporal dependencies and determined by biological mechanisms (e.g., a fixation is followed by a saccade and vice versa), which often results in high collinearity between the features^60^. From the class of machine learning models, we selected GBDT rather than other models like Support Vector Machines or Random Forest because of its ensemble approach. Ensemble methods can handle some degree of collinearity by partitioning the feature space into separate regions^61^. Previous research has demonstrated the suitability of GBDT models for spatial reasoning tasks involving geometrical objects, which are comparable to the task utilized in this study^62^.

Provided that the GBDT model classifies the conditions correctly, a Shapley Additive Explanations (SHAP) explainability approach can be applied^63^. The SHAP approach provides information on both global and local feature importance. Global feature importance ranks input features by their significance for accurate model predictions, identifying the most relevant features for differentiating between the experimental conditions. Local feature importance supplements this by providing additional information on the relationship between feature variables and target variables. It reveals which feature values were attributed to each condition and how effectively those values distinguish between conditions. These aspects complement statistical analyses and offer valuable insights into the relationship between eye movements and mental rotation processing.

## Results

### Mental rotation performance differences

All participants completed both experimental conditions (2D and 3D) in a block-wise randomized condition order. The mean values and standard deviations of all variables in each condition are depicted in Table 1. Further information about the distributions is presented in Supplementary Table S1. We used a non-parametric, paired Wilcoxon signed-rank test since some variables were not normally distributed. We report the Z statistics from two-tailed, paired tests with *p* values. Additionally, we applied a two-tailed, paired t-test and compared the results for skewed distributions (Supplementary Table S2).

**Table 1:** Mean values and standard deviations were aggregated on the participant level separately for each dimension ($n=54$).

Feature	2D ($M \pm SD$)	3D ($M \pm SD$)
Percentage solved correctly	$0.832\pm 0.105$	$0.882 \pm 0.101$
Reaction time (s)	$6.861\pm 3.583$	$6.076\pm 3.214$
Mean fixation duration (s)	$0.218\pm 0.025$	$0.216\pm 0.028$
Mean fixation rate (n/s)	$2.239\pm 0.266$	$2.301\pm 0.32$
Mean regressive fixation duration (s)	$0.142\pm 0.051$	$0.177\pm 0.042$
Equal fixation duration between figure (ratio)	$0.695\pm 0.086$	$0.721\pm 0.079$
Equal fixation duration within figures (ratio)	$0.187\pm 0.065$	$0.449\pm 0.084$
Strategy ratio ($\lessgtr 1$)	$1.488\pm 0.948$	$0.77\pm 0.292$
Mean saccade velocity ($^{\circ }/\textrm{s}$)	$239.186\pm 20.838$	$250.476\pm 22.439$
Mean saccades rate (n/s)	$2.016\pm 0.466$	$2.151\pm 0.451$
Mean pupil diameter (mm)	$0.039\pm 0.095$	$-0.096\pm 0.123$
Peak pupil diameter (mm)	$0.314\pm 0.101$	$0.416\pm 0.104$
Mean distance to figure (cm)	$88.599\pm 8.584$	$86.567\pm 10.21$
Mean head movement to the sides (cm)	$4.942\pm 3.595$	$5.713\pm 3.438$

Units are either seconds (s), number per second (n/s), a ratio between 0 and 1, or greater and smaller than 1 ($\lessgtr 1$), angle in degrees per second ($^{\circ }/\textrm{s}$), millimeters (mm), centimeters (cm), or centimeters per second (cm/s).

On average, participants spent 11.91 min in VR ($SD=3.65\;min$) without any breaks in between. In the 2D condition, participants solved $83.2\%$ of the stimuli correctly on average ($M=0.832$, $SD= 0.105$), while in the 3D condition, they solved $88.2\%$ correctly ($M=0.882$, $SD=0.101$). Participants achieved a significantly higher percentage of correct answers in the 3D condition ($Z=243$, $p=.001$) when comparing the 2D with the 3D condition in a two-tailed test. Participants exhibited a longer reaction time (in seconds, $M=6.861$, $SD=3.583$) in the 2D condition than in the 3D condition ($M=6.076$, $SD=3.214$). Based on a two-tailed test, reaction time differed significantly between the conditions ($Z=1168$, $p<0.001$). Details of the statistical analysis are shown in Table 2.

**Table 2:** Wilcoxon signed-rank tests comparing the 2D and 3D conditions ($n=54$).

Feature	Z	*p*	M diff	$95\%$ CI	Effect size
Percentage solved correctly	243	0.001	$-0.071\pm 0.014$	$[-0.089, -0.036]$	$-0.550$
Reaction time (s)	1168	$<0.001$	$0.644\pm 0.2$	$[0.317, 1.02]$	0.573
Mean fixation duration (s)	873	$>0.999$	$0.003\pm 0.003$	$[-0.002, 0.008]$	0.176
Mean fixation rate (n/s)	504	0.48	$-0.064\pm 0.029$	$[-0.125,-0.002]$	$-0.321$
Mean regressive fixation duration (s)	113	$$<0.001 $$	$$ -0.034\pm 0.005 $$	$$[-0.045, -0.024 ]$$	$$-0.848 $$
Equal fixation duration between figures (ratio)	477	0.276	$$-0.023\pm 0.01 $$	$$[-0.043, -0.003]$$	$$-0.358 $$
Equal fixation duration within figures (ratio)	1	$$ <0.001$$	$$-0.265\pm 0.011 $$	$$[ -0.286, -0.244]$$	$$ -0.999$$
Strategy ratio ($$\lessgtr 1$$)	1384	$$ <0.001$$	$$ 0.642\pm 0.106$$	$$[0.446, 0.868]$$	0.864
Mean saccade velocity ($$^{\circ }/\textrm{s}$$)	160	$$ <0.001$$	$$ -11.568\pm 1.692$$	$$[-15.208,-7.671 ]$$	$$ -0.785$$
Mean saccade rate (n/s)	339	0.012	$-0.148\pm 0.035$	$[-0.215,-0.07]$	$-0.543$
Mean pupil diameter (mm)	1438	$<0.001$	$0.134\pm 0.014$	$[0.104, 0.164]$	0.937
Peak pupil diameter (mm)	38	$$ <0.001$$	$$ -0.099\pm 0.011$$	$$[ -0.121, -0.079]$$	$$ -0.949$$
Mean distance to figure (cm)	1253	$<0.001$	$1.313\pm 0.681$	$[0.682, 2.114]$	0.688
Mean head movement to the sides (cm)	230	$<0.001$	$-0.618\pm 0.215$	$[-0.911,-0.368]$	$-0.69$

*p* values of all eye and head features were Bonferroni-corrected to account for multiple comparisons. A positive median difference value indicates a higher median value in the 2D condition (± standard error). The $95\%$ confidence interval for the median difference and rank biserial correlation effect size is reported. Units are either seconds (s), number per second (n/s), a ratio between 0 and 1, or greater and smaller than 1 ($\lessgtr 1$), angle in degrees per second ($^{\circ }/\textrm{s}$), millimeters (mm), centimeters (cm), or centimeters per second (cm/s).

To ensure that the differences in performance could not be attributed to sex differences, we performed additional statistical analyses to verify this. No sex differences were found in our study. This is consistent with previous research, which reported no sex differences in experiments conducted without time constraints^12,18^ or using less abstract stimulus materials^13,64^. Detailed statistics can be found in Supplementary Table S3.

We verified that the performance differences between 2D and 3D are not attributed to order effects. The average reaction time was always found to be higher in the 2D condition, regardless of the order. However, the differences were larger when the 2D condition was presented first. Similar results were observed for the percentage of correctly solved stimuli, for which the main differences were only present if the 2D condition was presented first. We also ensured that the sexes were equally distributed in both groups. The respective descriptive statistics can be found in Supplementary Table S4. In order to ensure that mental rotation in VR replicates expected differences, we provide additional descriptive statistics regarding reaction time and rotation angle for each condition separately in Supplementary Table S5.

To test for potential interaction effects between the experimental condition and the stimulus type (equal, mirrored, and structural), we conducted a multi-level regression analysis for each performance, eye, and head feature as the independent variable with condition and stimulus type as categorical independent variables. All analysis results and a model description can be found in Supplementary Table S10. Compared to equal figures, mirrored figures revealed a significantly lower percentage of correctly solved trials for the 3D condition. Structural figures, compared to equal figures, showed a significantly longer reaction time in the 3D condition.

### Statistical differences in eye and head movements

We tested for differences in all eye and head movement features between the two conditions using two-tailed, paired Wilcoxon signed-rank tests with aggregated values on the participant level. To consider multiple comparisons, all reported *p* values were Bonferroni-corrected before.

Regarding fixation-related features, we found no significant difference in the mean fixation duration ($$Z=873 $$, $$p>0.999$$) and the mean fixation rate ($$Z=504 $$, $$p=0.48$$). However, the mean fixation duration following a regressive saccade differed significantly between the conditions ($$Z=113 $$, $$p<0.001$$), with a higher duration in the 3D condition than in the 2D condition. The feature equal fixation duration between the figures showed no significant difference ($$Z=477$$, $$p=0.276$$) after correcting for multiple comparisons. The feature equal fixation duration within the figures showed a significant difference, with an equal distribution in the 3D condition ($$Z=1$$, $$p<0.001$$). The strategy ratio comparing the number of fixations within and between the figures showed a higher mean value for the 2D condition ($$Z=1384$$, $$p<0.001$$).

Regarding saccade-related features, there was a significant difference in mean saccade velocity ($Z=160$, $p<0.001$), with a higher mean value in the 3D condition. A higher mean saccade rate was found for the 3D condition ($Z=339$, $p=0.012$). Mean pupil diameter showed significantly higher values in the 2D condition ($Z=1438$, $p<0.001$), while peak pupil diameter was significantly lower in the 2D condition ($Z=38$, $p<0.001$). The mean distance to the figure and mean head movement to the sides differed significantly with closer distances to the figure in the 3D condition ($Z=1253$, $p<0.001$) and larger head movement to the sides in the 3D condition ($Z= 230$, $p<0.001$).

Regarding the interaction between the experimental condition and the stimulus type, three features showed significant interaction effects. When correcting for multiple comparisons, equal fixation duration within the figure showed lower values in mirrored figures (compared to equal ones) in the 3D condition. For structural figures (in comparison to equal ones), participants showed a higher mean saccade velocity and a lower mean saccade rate in the 3D condition (see Supplementary Tables S9 and S10).

### GBDT model capabilities

We trained a GBDT model to predict the experimental condition at the level of individual trials based only on eye and head movement features. $80\%$ of the data was used for training, with a random train-test split. In 100 iterations, predictions for the test set exhibited an average accuracy of 0.881 (with $SD=0.011$). The best-performing model had an accuracy of 0.918. False classifications were balanced between the two target conditions, with 27 trials misclassified as the 2D condition and 22 misclassified as the 3D condition. A confusion matrix for the best-performing model predictions is given in Table 3.

**Table 3:** Confusion matrix for 596 predicted trials (classified as either 2D or 3D) in the test set.

	2D labeled	3D labeled
2D predicted	267	22
3D predicted	27	280

Predictions of the best-performing GBDT model out of 100 iterations with a random 80:20 train-test split.

### Explainability results

**Figure 1:**
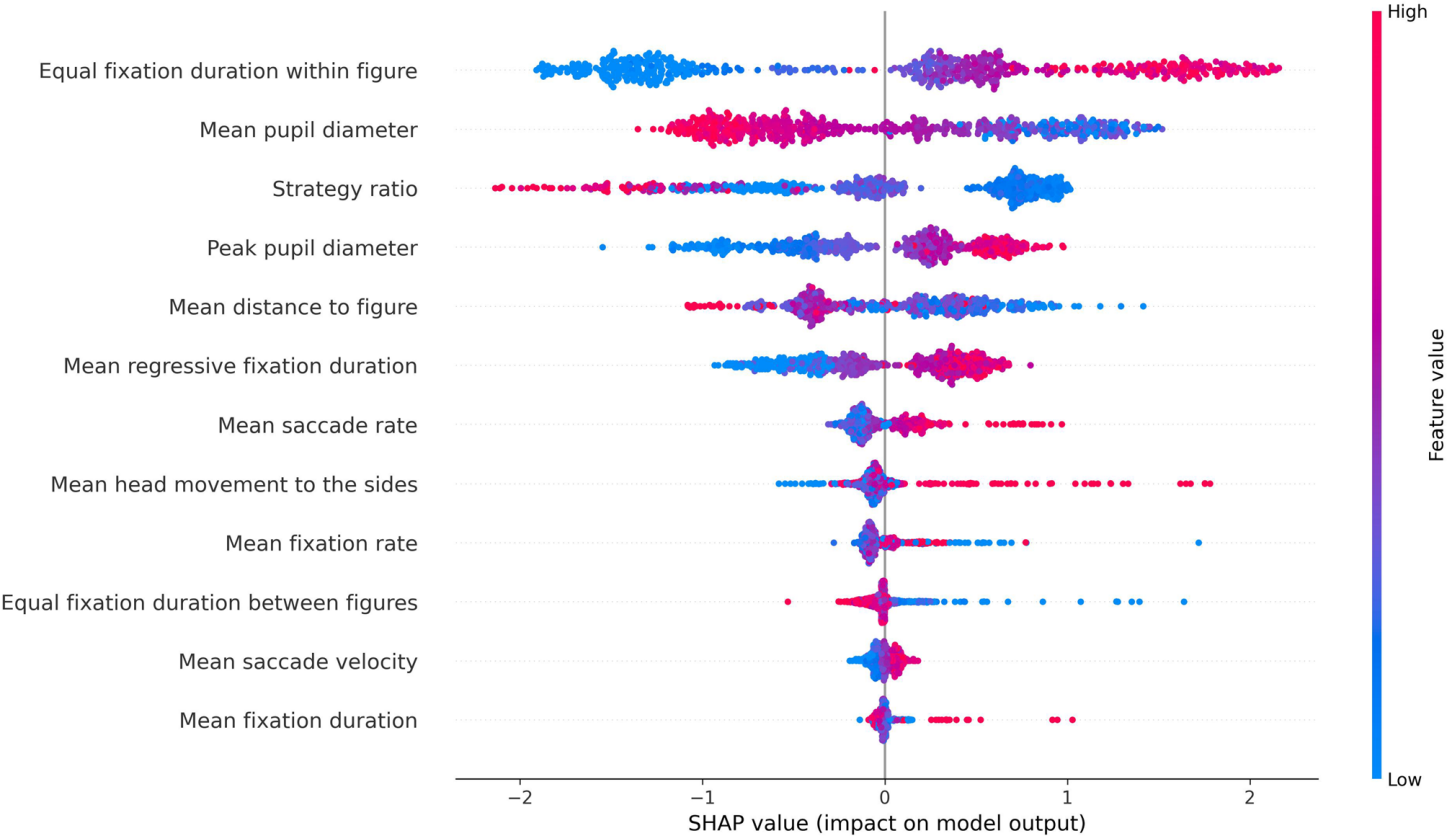
Summary plot of SHAP values for the GBDT model with the best performance out of 100 iterations (accuracy 0.918). Features are ordered according to their importance for the model’s predictions. The x-axis describes the model’s prediction certainty towards 2D (left side) and 3D (right side). Data points are predicted trials. The red color indicates that the data point has a high value for the feature, and the blue color indicates that the data point has a low value for that feature.

We applied the SHAP Tree Explainer^63^ to the best-performing model. Equal fixation duration within the figure was rated the most important feature for the GBDT model, with smaller values leading to predicting the 2D condition and larger values the 3D condition. The second most important feature was mean pupil diameter, with a higher mean pupil diameter leading to predicting the 2D condition. The third most important feature was the strategy ratio, with higher values leading to predicting the 2D condition and low values the 3D condition. Peak pupil diameter was identified as the fourth most important feature, with the opposite tendency as mean pupil diameter. A higher peak pupil diameter led to predicting the 3D condition. Mean distance two the figure (5th) showed a tendency to predict the 2D condition for higher values. However, there is higher variability in feature values in both conditions. For the following three features, mean regressive fixation duration (6th), mean saccade rate (7th), and mean head movement to the sides (8th), the model showed a tendency to associate higher values with the 3D condition. The remaining features exhibited little importance for model prediction or no clear tendency towards one condition or the other. The results are visualized in Fig. 1. Based on the additional analysis for multi-collinearity (see Supplementary Table S6), we found no high correlations between the individual features. A larger negative correlation was found between mean saccade rate and mean fixation duration ($r=-0.39$) and between mean saccade rate and strategy ratio ($r=-0.31$).

## Discussion

This study used a VR laboratory to test mental rotation, presenting Shepard and Metzler^7^ stimuli in a controlled yet ecologically valid environment. Specifically, our study investigated whether the mode of presentation (i.e., pictorial 2D or visual 3D figures) evoked differences in visual processing during task solving and affected participants’ performance. Participants’ mental rotation test performance differed significantly between the two presented conditions, with higher accuracy and shorter reaction time in the 3D than in the 2D condition. These findings are in line with previous research reporting better performance for 3D figures^31,65^. We argued that the direct encoding of visual figures would allow for faster and easier processing in the 3D condition, leading to a decrease in response time. In addition, we argued that access to depth information via binocular disparity and motion parallax would enhance stimulus perception and facilitate the transformation and comparison of visual figures. These factors could have led to improved performance on mental rotation tasks in the 3D condition. In addition, motion parallax in the 3D condition provided the opportunity to use head movements to change perspective (e.g., egocentric perspective taking). In combination with easier perception of the geometric structure of the figures, this could have led to a more holistic processing of the stimuli.

We analyzed eye and head movement information to substantiate these assumptions. We argued that the changes introduced by the mode of presentation and their effect on stimulus processing and mental rotation strategies can be investigated by analyzing participants’ visual behavior. The successful training of the GBDT model indicated that the eye and head movement features provided valuable information to distinguish between the two conditions. Statistical analysis, as well as SHAP values, discriminated different eye and head movement patterns in both conditions.

Overall, our results indicate that the additional information provided by motion parallax led to more pronounced head movement to the sides and a closer inspection of the visual 3D figures. In turn, directly inspecting hidden parts of the depicted figures by changing perspective could have resulted in a less ambiguous perception of the figure^66^.

At a more detailed level, our findings suggest that fixation patterns in the 2D condition related more strongly to the first processing step of encoding and searching, while patterns in the 3D condition were related to the step of transformation and comparison. Xue et al.^33^ found that the first step was associated with more fixations on particular segments of the figures. In contrast, the second step showed a more equal distribution of fixations across all segments of the figures. The SHAP value analysis indicated that the two conditions mostly differed in fixation duration within the figures. A less equal distribution within the figures, which implies longer fixations on particular segments, was found in the 2D condition. This supports the claim that the availability of depth information through motion parallax and binocular disparity accelerated the initial encoding of the visual figures and allowed participants to move more quickly to subsequent steps. In the same vein, a lower saccade velocity was found in the 2D condition, indicating more saccades within particular segments of the figures. However, in the 3D condition, participants moved their heads, on average, closer to the figures. This increases the saccade amplitude since the distances between and within figures become larger, which in turn increases saccade velocity^67^. The inverse correlation of $r=-0.24$ between saccade velocity and distance to the figure indicates that, at least to some degree, saccade velocity is affected by participants’ head movements (see Supplementary Table S6).

Furthermore, the mean pupil diameter was larger in the 2D than in the 3D condition, while the peak pupil diameter was smaller in the 2D condition than in the 3D condition. The larger mean pupil diameter as an indicator of tonic activity could imply higher task difficulty and lower task utility in the 2D condition. This can be further supported by the lower saccade rate in the 2D condition. A decreasing saccade rate was previously associated with an increase in task difficulty^68^. In contrast, the smaller peak pupil diameter as an indicator of phasic activity could imply lower engagement and less task-relevant exploitation of the 2D task. These results provide further evidence that the first step of encoding might be more demanding for the pictorial 2D figures, and additional information due to head movement might have facilitated task-relevant exploitation. Moreover, a shorter average fixation duration after a regressive saccade in the 2D condition could indicate a need for more information retrieval when trying to maintain a 3D mental model of the figures in mind.

At the same time, our study findings indicate that presentation mode might confound previous research on individuals’ strategies for solving mental rotation tasks. The presentation of 2D figures was more strongly related to features indicating a piecemeal strategy than the presentation of 3D figures. This was implied by differences in the strategy ratio used to distinguish between holistic and piecemeal strategies^35,42^. Our results showed that participants in the 2D condition moved their gaze more frequently within a figure and switched fewer times between figures than in the 3D condition. Consequently, one might assume that the 2D presentation mode could evoke piecemeal processing. In this case, however, the strategy ratio not only reflected the way in which the figures were compared but could also be affected by differences in the first step of encoding the figures. Our results clearly speak to the relevance of different processing steps, which need to be considered more carefully in future research. For instance, the reason why mental rotation seems to be easier with more natural stimuli^64^ could be that encoding figure information is less demanding.

Results of the interaction analysis indicated that a faster encoding of the figure and more holistic processing in 3D were associated with some costs. Participants made relatively more mistakes with mirrored stimuli in the 3D condition, and took a relatively longer time for structural figures compared to equal figures. In addition, eye movement features showed that participants took more time investigating specific parts of the figure for structural stimuli compared to equal stimuli in the 3D condition. When searching for the misaligned segment in structurally different stimuli, participants potentially switched from a holistic strategy to a piecemeal strategy, which in turn resulted in longer reaction time with this stimulus type.

In sum, our study showed how eye and head movements could be used to investigate systematic differences in stimulus processing and mental rotation strategies across different modes of presentation. However, we are also aware of the potential limitations of the present study. Although we were able to show that the mode of presentation causes a difference in processing, we cannot determine, for example, in which of the steps individuals with high and low abilities differ. Furthermore, our results suggest that the strategies used are related to the mode of presentation. Although we identified strategies using a common indicator^35,42^, future studies should expand on this using more elaborate methods, such as ones allowing for time-dependent analyses. Moreover, the accuracy of the VR eye tracker was a technical limitation of our study. Previous studies using the same eye-tracking device have reported lower gaze accuracy in the outer field of view^69^. By using the VIVE Sense Eye and Facial Tracking SDK (Software Development Kit) to capture eye-tracking data in the Unreal engine, the frame rate of the eye tracker was adjusted to the lower refresh rate of the game engine. Therefore, our eye tracking in VR did not provide the same spatial and temporal resolution as remote eye trackers. There was also a limitation regarding the usability of head-mounted displays. Although we used the latest VR devices in our experiment, the participants had the added weight of the HMD on their heads, and we had to connect the HMD device to the computer with a cable. This limited the participants’ freedom of movement to some degree and may have affected the extent of their head movement and natural exploration. Another limitation concerns a possible confounding effect between head movement and fixations due to the vestibular eye reflex. This reflex stabilizes vision when fixating during head movement and could, therefore, compromise fixation-related features due to the influence of automated adjustments^70,71^. The bivariate correlations between $r=-0.07$ and $r=-0.11$ revealed only small relationships between both head movement and all fixation-related features for both the 2D and 3D conditions on the level of individual trials (see Supplementary Table S7 and S8). While one cannot rule out the effect of vestibular eye reflex on fixation-related features, the study findings indicated a similarly small influence of the vestibular eye reflex on fixations in both conditions.

Despite these limitations, VR proved to be a useful tool to test mental rotation ability in an ecologically valid but controlled virtual environment. We made use of integrated eye tracking to learn more about the impact of presentation modes on stimulus processing and mental rotation strategies when solving Shepard and Metzler stimuli. Our results indicated that mental rotation places different demands on different processing steps when processing pictorial or visual figures. The demands that pictorial 2D figures place on participants, from encoding to rotating the figures, seem to be ameliorated by the provision of additional visual information. More importantly, our results suggest that 2D figures evoke piecemeal analytic strategies in mental rotation tasks. This, in turn, leads to the question of whether piecemeal processing tells us more about the ability to create and maintain 3D representations of 2D images than it does about the ability to rotate one 3D figure into another.

## Methods

### Participants and procedure

During data collection, 66 university students participated in the experiment. Due to missing eye-tracking data, we had to exclude 12 participants. Data from 54 participants remained for the analysis. In the remaining sample, 33 participants stated their sex as female and 21 as male. Participants’ average age was 24.02 ($SD = 7.24$), and 35 of them needed no vision correction, while 19 wore glasses or contact lenses.

The experiment took place in an experimental lab at a university building. After providing written informed consent to participate, participants completed a pre-questionnaire. The pre-questionnaire asked for socio-demographic and personal background information. Before using the VR, participants were informed about the functionality of the device and a five-point calibration was performed with the integrated eye tracker. After that, participants conducted the mental rotation test in VR. In the test, participants had to go through 60 stimuli one after another. Each stimulus displayed two Shepard and Metzler figures, for which participants had to respond whether they were equal or unequal using the handheld controllers^7^. 30 of the stimuli were presented on a virtual screen, replicating a classical computerized Shepard and Metzler test (2D condition). The other 30 stimuli were displayed as 3D-rendered objects floating above a table (3D condition). Participants were randomly assigned to first see all 2D or all 3D stimuli. Randomization was used to balance out any kind of sequence effect. Out of the 54 participants, 31 saw the 2D experimental condition first, and 23 saw the 3D experimental condition first. No time limit was set for completing the tasks. After completing the experiment, participants received compensation of 10€. The total experiment did not exceed 1 h, and the VR session did not exceed 30 min. To complete both VR conditions, participants spent, on average, 11.91 min in VR ($SD=3.65\;min$) without any breaks in between. The study was approved by the ethics committee of the Leibniz-Institut für Wissensmedien in Tübingen in accordance with the Declaration of Helsinki.

### Experiment design

#### VR environment

**Figure 2:**
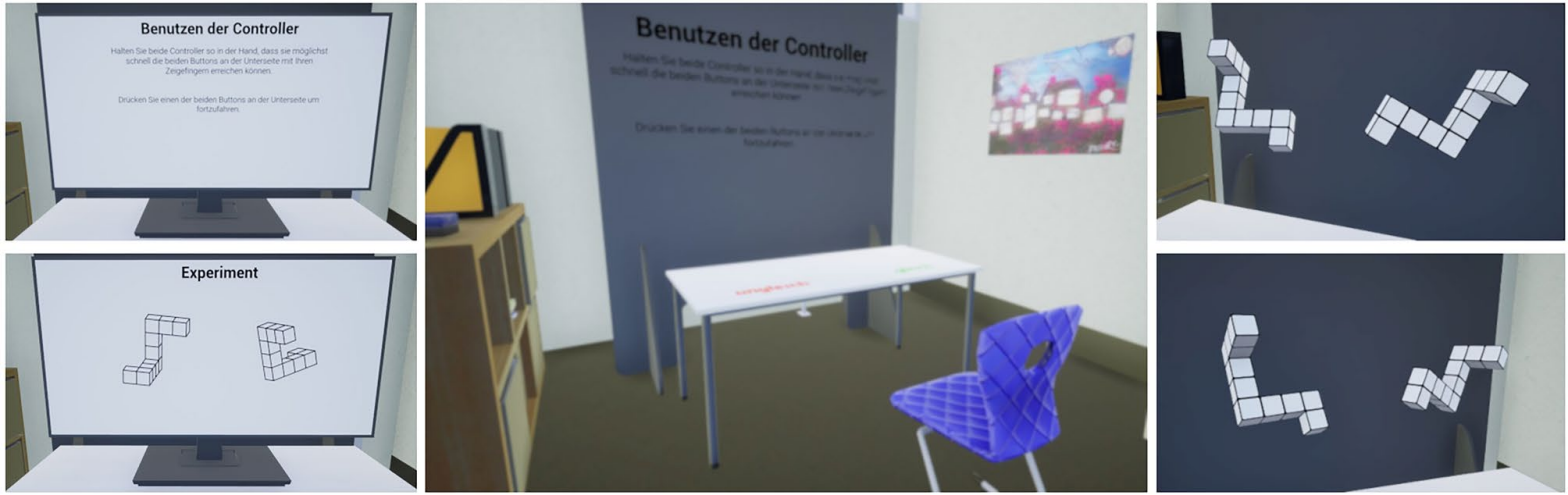
Images taken from our VR environment show the virtual experiment room as well as example stimuli from the 2D and 3D conditions embedded in the environment.

The VR environment was designed and implemented in the game engine Unreal Engine 2.23.1^72^. Participants sat on a real chair in the experiment room and entered a realistically designed virtual experiment room, where they also sat on a virtual chair in front of a desk (see Fig. 2). Before the start of the mental rotation task, instructions were shown in the 3D condition on a virtual blackboard located behind the experimental table in the participants’ direct line of sight, whereas for the 2D condition, the instructions were presented on the virtual screen display. Participants were instructed to solve the tasks correctly and as quickly as possible. Additionally, participants completed one equal and one unequal example stimulus pair, after which they received feedback on whether the examples were solved correctly or incorrectly. After they responded with the controllers, a text was displayed on the blackboard or the screen. The stimuli appeared at a distance of 85 cm from the participants. For the 2D condition, the stimulus material appeared on a virtual computer screen placed on the desk. During the 2D condition, the screen was visible at all times; only in the center of the screen did the figures appear and disappear. In the 3D condition, the stimulus material appeared floating above the table. The 3D figures were rendered as 3D objects in the environment, which allows the figures to be viewed from all perspectives. The distance to the center of the 3D figures was the same as the distance to the screen in the 2D condition. The figures were also placed at the same height in both conditions. Before a stimulus appeared, a visual 3-second countdown marked the start of the trial. Participants then decided whether figures were equal or unequal and indicated their response by clicking the right or left controller in their hands (left = unequal, right = equal). Instructions on using the controllers were displayed on the table in front of them.

#### Stimulus material

Our mental rotation stimuli were replications of the original test material by Shepard and Metzler^7^. The 2D mental rotation test was designed as a computerized version and presented on the VR virtual screen. For the immersive 3D condition, the original test material was rendered as 3D objects in VR. In both conditions, each stimulus consisted of two geometrical figures presented next to each other.

**Figure 3:**
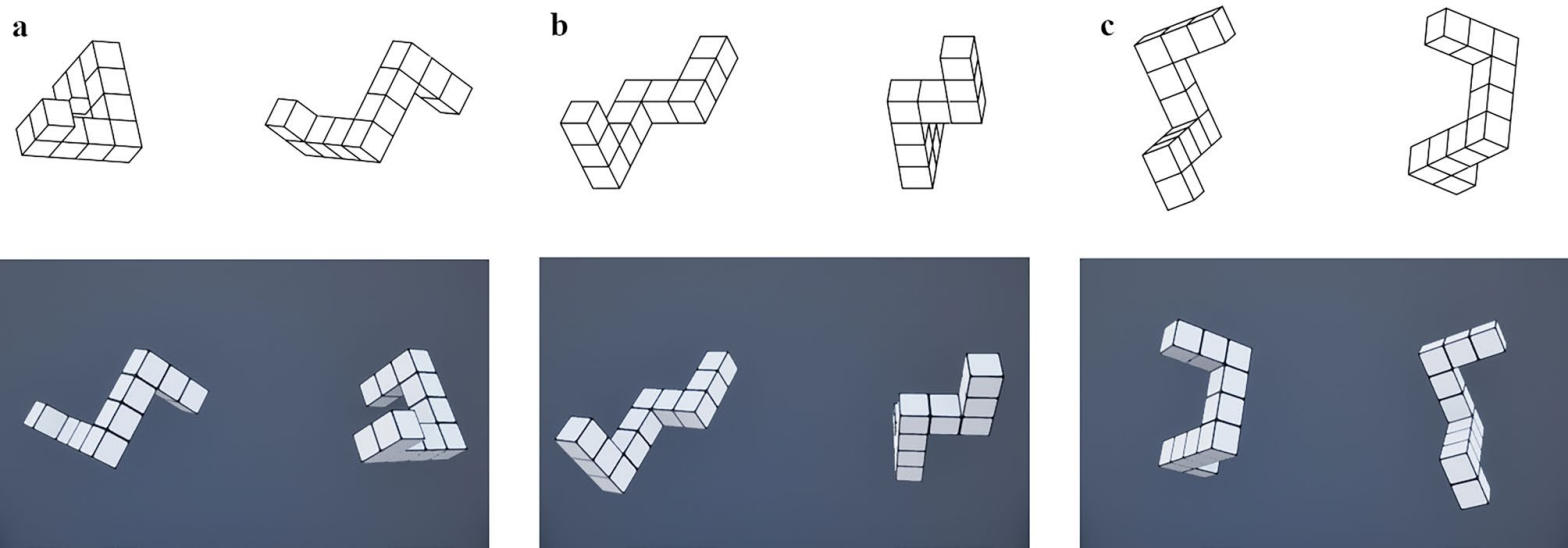
Examples of our stimulus material with three different types of mental rotation stimuli for 2D (top) and 3D (bottom). Figure sides (left or right) were randomly switched between 2D and 3D to avoid memory effects. The 3D images are screenshots of the VR environment. (**a**) Equal pairs. (**b**) Mirrored unequal pairs. (c) Structural unequal pairs.

One figure was always a true-to-perspective replication of the Shepard and Metzler material used in previous experiments^65,73^. These figures and their form of presentation have been used in various studies and provide a reliable and valid basis for our experimental material^2,13,74,75^. These stimuli were created by rotating and combining ten base figures^76^. Each base figure was a 3D geometrical object composed of 10 equally sized cubes appended to each other. The cubes formed four segments pointing in different orthogonal directions. This resulted in three possible combinations for the figure pairs: Either they were the same (equal pairs) or not the same (unequal). If unequal figure pairs had the same number of cubes per segment, but one figure was a mirrored reflection of the other, we called it an unequal mirrored pair. If the unequal figure pairs were similar, except one segment pointed in a different direction, we called it an unequal structural pair. Examples for all three stimulus types are depicted in Fig. 3. Variation in task difficulty was induced by rotating one figure along its vertical axis by either 40, 80, 120, or 160 degrees while keeping the other figure in place. Ergo, each stimulus showed one of the four rotation angles. Due to incorrect visual displays, two stimuli had to be removed from the experiment since different figures were presented in the two conditions. This resulted in 28 stimuli used for data analysis. For all 28 stimuli, we ensured a relatively equal distribution of all four displacement angles and an equal number of equal and unequal trials. The distribution of stimulus characteristics can be found in Table 4.

We rendered the figures using the 3D modeling tool Blender^77^. For the 2D condition, we took snapshots in Blender. For the 3D condition, we imported the 3D models into the VR environment. The 3D models could then be displayed, positioned, and rotated there. To compare the 2D and 3D conditions, we used the same combination of base figures and the same rotation angles in each stimulus. The figures’ rotation direction and left-right position were varied to reduce memory effects.

**Table 4:** Characterization of presented stimuli according to their rotation angle (in degree) and their stimulus type.

Characteristic	Category	Number
Angular disparity	40	9
Angular disparity	80	5
Angular disparity	120	7
Angular disparity	160	7
Stimulus type	Equal	14
Stimulus type	Mirrored	9
Stimulus type	Structural	5

#### Apparatus

An HTC Vive Pro Eye and its integrated Tobii eye tracker were used for the VR experiment. The Dual OLED displays inside the HMD provided a combined resolution of $2880 \times 1600$ pixels, with a refresh rate of 90 Hz. The integrated Tobii eye tracker had a refresh rate of 120 Hz and a trackable FOV of $110^{\circ }$, with a self-reported accuracy of $0.5-1.1^{\circ }$ within a $20^{\circ }$ FOV^78^. We ran the VR experiment on a desktop computer using an Intel Core i7 processor with a base frequency of 3.20GHz, 32 GB RAM, and an NVIDIA GeForce GTX 1080 graphic card.

#### Data collection

While participants used the VR, our data collection pipeline saved stimulus, eye-tracking, and HMD-movement information at each time point, marked with a timestamp. A time point is determined by the VR device’s frame rate and the PC’s rendering performance. The average frame update rate for all VR runs was 27.31 ms ($SD = 3.36$  ms), which translates to 36.61 frames per second. For all experiment runs, the average standard deviation was 6.14 ms. At each frame, we collected eye-tracking data from the Tobii eye tracker, as well as head movement and head rotation. We also noted which stimulus was being presented and if the controllers were being clicked.

We used gaze ray-casting to obtain the 3D gaze points (the location where the eye gaze focuses in the 3D environment). Gaze ray-casting is a method to determine where participants are looking within the scene. For this method, the participant’s gaze vector is forwarded as a ray into the environment to see what it intersects with^79,80^. In our experiment, this gaze intersection was either the virtual screen in the 2D condition or an invisible surface for the 3D condition at the same position.

### Data processing

#### Data cleaning and pre-processing

After cutting the instructions and tutorial at the beginning of the experiment, we dropped participants with an average tracking ratio below $80\%$ in the raw left and right pupil diameter variables. Since we wanted to compare both conditions (2D and 3D) for each participant, sessions in which only one of the two conditions showed a low tracking ratio also had to be excluded.

The integrated eye tracker already marks erroneous eye detections in the gaze direction variables, which we used to identify missing values. Since blinks are usually not longer than 500 ms^81^, only intervals up to 500 ms were considered blinks. We needed to detect blinks to correct for artifacts and outliers around blink events^82,83^. To remove possible blink-induced outliers, we omitted one additional data point around blink intervals, meaning that based on our frame rate, on average, 27 ms around blinks was missing.

Combined pupil diameter was calculated as the arithmetic mean of the pupil diameter variables for both eyes. A subtractive baseline correction was performed separately for each individual trial. We obtained individual baselines by calculating the median over the 3-second countdown before the stimulus appeared. The values of the combined pupil diameter during the stimulus intervals were corrected by the baseline measured shortly before. This ensured that potential lighting changes, different background contrasts, or increased fatigue were considered and controlled for^84^.

We calculated gaze angular velocity from the experiment data as the change in gaze angle between consecutive points (in degrees per second). The mean distance to the figure was calculated by taking the Euclidean distance between the participant’s head location and the midpoint of the stimulus. Additionally, for the 3D condition, we calculated 2D gaze points on an imaginary plane. This plane was set to the same position as the screen in the 2D condition.

#### Fixation and saccade detection

We applied a combination of a velocity identification threshold (I-VT) and a dispersion identification threshold (I-DT) algorithm for the 2D gaze points^85^. I-VT could be used to detect fixations during stable head movements. However, it was possible to fixate on one spot while rotating one’s head around the figure. Because we assumed differences in head movements between the conditions, this would cause artificial differences between conditions. To address this problem of free head movement, we additionally used an I-DT fixation detection algorithm to detect unidentified fixation during periods of head movement.

The I-VT algorithm detected a fixation if the head velocity was $< 7^{\circ }/\textrm{s}$ and the gaze velocity was $< 30^{\circ }/\textrm{s}$. We applied the thresholds for each successive pair of data points by dividing the velocity of the gaze or head angles by the time difference between the points. We considered intervals with a duration between 100 and 700 ms as fixations. We labeled data points as saccades if the gaze velocity was $>60^{\circ }/\textrm{s}$ and its duration was below 80 ms. Thresholds for the I-VT algorithm to detect fixation were set conservatively^86^. For the I-DT algorithm, a dispersion threshold of $2^{\circ }$ and a minimum duration threshold of 100 ms were set. To calculate the dispersion, the angle from one data point to another was used, considering the average distance of the participant to the screen or the imaginary surface. Table 5 shows an overview of the parameters.

Similar threshold parameters for both algorithms have been used in other VR and non-VR studies^85–87^. The final number of fixations was then formed as a union of both algorithms. We calculated the fixation midpoint for each fixation interval as the centroid point.

**Table 5:** Threshold parameters for detecting fixations and saccades of the velocity and dispersion identification algorithms.

	I-DT fixations	I-VT fixation	I-VT saccades
Head velocity ($v_h$)	–	$v_h<7^{\circ }/\textrm{s}$	–
Gaze velocity ($v_g$)	–	$v_g < 30^{\circ }/\textrm{s}$	$v_g > 60^{\circ }/\textrm{s}$
Gaze dispersion ($d_g$)	$d_g < 2^{\circ }$	–	–
Duration ($\Delta$)	$\Delta > 100\,\textrm{ms}$	$100\,\textrm{ms}< \Delta < 700\,\textrm{ms}$	$\Delta < 80\,\textrm{ms}$

#### Gaze target information

To calculate features that encode spatial information, for example, on which objects participants fixated, we had to apply further processing steps. This procedure was used to determine whether the fixation location was on or close to one of the figures for each fixation event. If this was the case, the fixation was marked as being on a figure (left or right) and on a specific segment of this figure (inner or outer segment).

Gaze information collected from the VR eye tracker only provides local information about the gaze direction. This means the coordinate system is independent of head movement and head location. The local gaze direction must first be cast into the virtual space by a so-called gaze ray-casting method^80,88^ to get the gaze direction in the virtual space. To find out which object the gaze landed on, the following steps had to be applied. After fixation events are detected, the centers of the fixations hit certain locations in the virtual environment. These locations, also called gaze targets, could either be on the mental rotation figures, close to them, or somewhere else.

**Figure 4:**
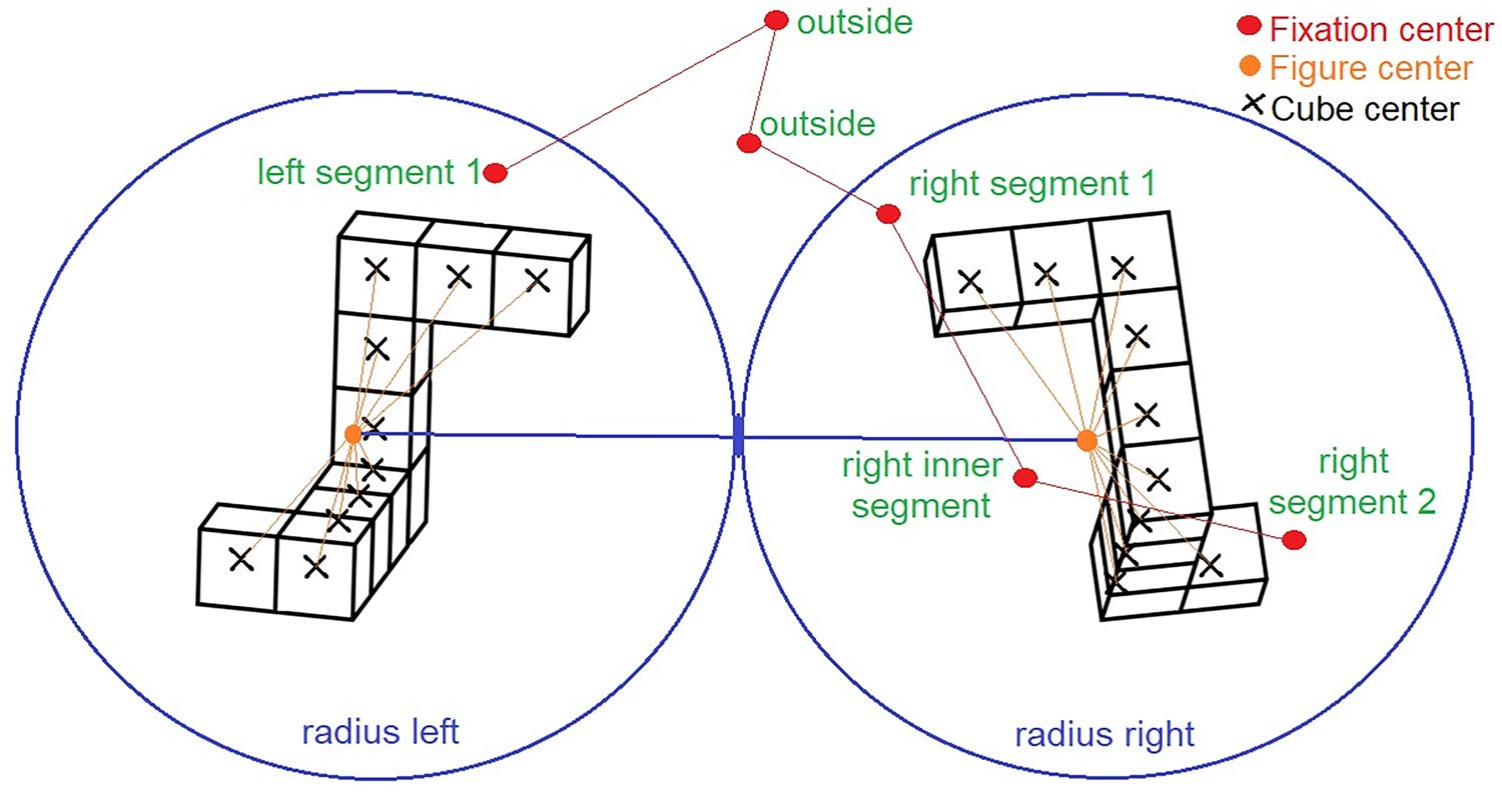
A not-true-to-scale illustration of the processing steps involved in finding the closest segments of the figures for each fixation center.

Lower accuracy and precision of the HMD produced an offset between the fixation location and the figures. However, we wanted to obtain the most relevant gaze target information. Therefore, fixation locations on a figure, as well as close to a figure, were assigned to that figure. More precisely, for each gaze location, we checked which figure cubes were located close to it. We then checked whether these cubes corresponded to the same segment of the same figure. If the majority of cubes belonged to one segment of one figure, we labeled the fixation location to be on this particular segment. To only assign fixation locations close to the figures, we additionally checked the distance between the fixation locations and the figure centers. If the distance was larger than a radius, we rejected the fixation locations and labeled them as not being on a figure. The radius was obtained by calculating the distance between both figure centers. We calculated the figure centers as the centroid point of all cube midpoints for one figure. Cube midpoints in the 2D condition were based on manual annotations done by a student assistant with the Computer Vision Annotation Tool https://github.com/opencv/cvat. (Retrieved 9/21/2023). To check if all manual annotations were correct, we reconstructed figure plots from the annotation data. Cube midpoints of the 3D figures were collected in the VR environment. An illustration of the process is shown in Fig. 4.

### Feature aggregation

#### Performance measures and condition

Out of the 3024 total presented stimuli (28 stimuli x 54 participants), we needed to remove 46 of these trials due to missing values on at least one feature variable. 2978 trials could be used for the analysis. For each variable, we aggregated the values using the arithmetic mean over all of a person’s trials in the 2D and 3D conditions separately.

Reaction time for each trial was calculated using the timestamps in the data. Participants’ controller responses were also tracked during the experiment and could be used in combination with a stimulus number to determine a correct or incorrect answer. The experimental data also stored the target variable (2D or 3D).

#### Eye movement features

Based on the processed experiment data, all eye-movement features were calculated for each stimulus interval separately. For a clearer overview, a description of each feature with the corresponding unit and its calculation is given in Table 6. We focussed on calculating measures shown to be less affected by sampling errors given a lower sampling frequency (e.g., fixation duration, fixation rate, and saccade rate) and ignored features like saccade duration^89,90^. Special attention was paid to the selection of the event detection algorithms to increase reliability by combining two detection algorithms (I-VT and I-DT). We also tried to average out potential outliers by averaging over longer time intervals (Mean fixation duration or mean pupil diameter). To reduce noise and the influence of artifacts on peak pupil diameter, maximum and minimum were only taken within an 80% confidence interval.

**Table 6:** Descriptions of all calculated eye-movement features per stimulus interval.

Name	Description
Mean fixation duration	Average durations in seconds of all fixations within a stimulus interval
Mean fixation rate	Average over the number of fixations per second
Mean regressive fixation duration	Average duration in seconds of all fixations after a regressive saccade
Equal fixation duration between figures	Ratio of the distribution of duration between the figures. Values close to zero indicate that most fixation duration is only on one figure. Values close to one indicate equal fixation duration on both figures (left and right)
Equal fixation duration within figure	Ratio of the distribution of the fixation duration on the whole figure. A value close to zero indicates the most fixation duration on one part of the figures (outer or inner part). A value close to 1 means equal distribution on the outer and inner parts of the figures
Strategy ratio	Ratio of the number of fixations within the figure divided by the number of saccades between the figure. The number of saccades started as one for the first look at one figure
Mean saccade velocity	Average over velocities in gaze angle (degree per second) between consecutive time points
Mean saccade rate	Average over the number of saccades per second
Mean pupil diameter	Average of all corrected pupil diameter values in millimeters within a stimulus interval.
Peak pupil diameter	Distance between the lowest and highest corrected pupil diameter values in millimeters within an 80% confidence interval of all pupil diameter values within a stimulus interval
Mean distance to figure	Average Euclidean distance in centimeters from the participants’ head to the figure midpoint
Mean head movement to the sides	Average absolute head movement on the lateral axis in centimeters from the starting position of the participant’s head

### Data analysis

#### Statistical analysis

The differences between the conditions in some variables were not normally distributed. Thus, we applied a non-parametric, two-tailed, paired Wilcoxon signed-rank test to compare the percentage of correct answers and reaction times between the conditions. We applied the same test for the eye-movement features but corrected the *p* values according to Bonferroni’s correction. Moreover, we applied a two-tailed, paired t-test for additional verification. The test showed no considerable differences in the *p* values for any variables.

#### Machine learning model

We used a Gradient Boosting Decision Tree (GBDT) classification algorithm to classify the experimental condition since this model had shown high predictive performance in studies with similar data and tasks^62^. Before training the model, we split our data randomly into training and test sets using an 80 to 20 ratio. To increase the reliability of the model performance, we applied a random train-test-split cross-validation with 100 iterations. We trained a GBDT model with eye-movement features at the individual trial level. The model was trained using default hyper-parameters for the Gradient Boosting Classifier from the scikit-learn Python package^91^. We used the 2D or 3D experimental conditions as targets in a binary classification task.

#### Metrics to evaluate model performance

The within-subject design of the study resulted in almost-balanced sample classes. For the binary classification task (2D and 3D conditions), true positive (TP) cases were correct classifications to the 2D condition, and true negative (TN) cases were correct classifications to the 3D condition (and vice versa for false positives (FP) and false negatives (FN)). The performance metric accuracy was calculated as

$$\begin{aligned} accuracy = \frac{\text {Number of TP} + \text {Number of TN}}{\text {Total Number of Cases}} \end{aligned}$$

We report the mean and standard deviation for the accuracy scores over all 100 iterations and for the best-performing model.

#### Explainability approach

To see how the model uses the measures for prediction, we applied a post-hoc explainability approach using Shapley Additive Explanations (SHAP). Specifically, we used the TreeExplainer algorithm, which computes tractable optimal local explanations and builds on classical game-theoretic Shapley values^63^. Unlike other explainability approaches, which provide information about the global importance of input features, this algorithm computes the local feature importance for each sample. This means we could obtain the importance value for each feature for each classified sample. If a feature exhibited a positive importance value, it drove the model classification towards the positive class and vice versa. The greater the absolute value, the greater its impact on the classification decision. Hence, the overall importance of a feature for classification can be measured by taking the average of the absolute importance values across all samples. Results for local feature importance in the best-performing models are reported in a set of beeswarm plots. The order of the features in the plot represented their overall importance, and each dot displayed the importance and feature value for one sample. Correlated features confound the interpretation of SHAP feature importance for decision tree algorithms. If two features are highly correlated, the algorithm might choose only one feature for prediction and ignore the other completely. Therefore, we checked for multi-collinearity by looking at all measures’ pairwise Pearson correlations.

## Supplementary Information

Supplementary Information.

## Data Availability

The datasets generated and/or analyzed during the current study are available in the osf.io repository, https://osf.io/vjzmf/?view_only=63de2d2576f04f7cb8059d9669af36c9
