# Supplementary material for: The impact of presentation modes on mental rotation processing: a comparative analysis of eye movements and performance
Source: Sci Rep. 2024 May 29;14:12329. doi: 10.1038/s41598-024-60370-6 (PMC11589343; doi:10.1038/s41598-024-60370-6)
Supplement: Supplementary file 1 — Supplementary Information. [file 41598_2024_60370_MOESM1_ESM.pdf]

Supplementary Information

Supplementary Table S1: Table of additional descriptive statistics

Variable	Mean	Median	[Min, Max]	Skewness	Kurtosis	SE
Percentage solved correctly 2D	0.832 ± 0.105	0.857 ± 0.106	$[0.500, 1.000]$	-0.962	0.718	0.014
Percentage solved correctly 3D	0.882 ± 0.101	0.893 ± 0.106	$[0.607, 1.000]$	-1.011	0.322	0.014
Reaction time (s) 2D	6.861 ± 3.583	5.834 ± 1.968	$[1.640, 16.825]$	1.230	0.800	0.488
Reaction time (s) 3D	6.076 ± 3.214	4.938 ± 2.030	$[2.359, 18.539]$	1.613	2.958	0.437
Mean fixation duration (s) 2D	0.218 ± 0.025	0.215 ± 0.026	$[0.166, 0.270]$	0.167	-0.908	0.003
Mean fixation duration (s) 3D	0.216 ± 0.028	0.214 ± 0.024	$[0.174, 0.311]$	1.050	1.473	0.004
Mean fixation rate (n/s) 2D	2.239 ± 0.266	2.194 ± 0.241	$[1.743, 2.900]$	0.474	-0.304	0.036
Mean fixation rate (n/s) 3D	2.301 ± 0.320	2.269 ± 0.301	$[1.699, 3.051]$	0.274	-0.537	0.044
Mean regressive fixation duration (s) 2D	0.142 ± 0.051	0.139 ± 0.054	$[0.042, 0.249]$	0.069	-0.710	0.007
Mean regressive fixation duration (s) 3D	0.177 ± 0.042	0.179 ± 0.047	$[0.105, 0.284]$	0.293	-0.535	0.006
Equal fixation duration between figure (ratio) 2D	0.695 ± 0.086	0.695 ± 0.094	$[0.447, 0.866]$	-0.500	0.214	0.012
Equal fixation duration between figure (ratio) 3D	0.721 ± 0.079	0.730 ± 0.082	$[0.437, 0.857]$	-0.849	1.228	0.011
Equal fixation duration within figures (ratio) 2D	0.187 ± 0.065	0.187 ± 0.055	$[0.020, 0.340]$	-0.199	0.394	0.009
Equal fixation duration within figures (ratio) 3D	0.449 ± 0.084	0.465 ± 0.086	$[0.237, 0.632]$	-0.328	-0.105	0.011
Strategy ratio ($\lesseqgtr 1$) 2D	1.488 ± 0.948	1.321 ± 0.841	$[0.223, 4.357]$	0.880	0.204	0.129
Strategy ratio ($\lesseqgtr 1$) 3D	0.770 ± 0.292	0.722 ± 0.304	$[0.364, 1.585]$	0.869	0.221	0.040
Mean saccade velocity ($°/s$) 2D	239.18 ± 20.83	235.71 ± 17.46	$[198.82, 295.22]$	0.598	0.109	2.836
Mean saccade velocity ($°/s$) 3D	250.47 ± 22.43	249.63 ± 20.97	$[203.63, 315.91]$	0.415	0.275	3.054
Mean saccade rate (n/s) 2D	2.016 ± 0.466	1.992 ± 0.485	$[1.309, 3.280]$	0.567	-0.220	0.063
Mean saccade rate (n/s) 3D	2.151 ± 0.451	2.132 ± 0.481	$[1.359, 3.169]$	0.248	-0.712	0.061
Mean pupil diameter (mm) 2D	0.039 ± 0.095	0.038 ± 0.077	$[-0.201, 0.236]$	-0.568	0.479	0.013
Mean pupil diameter (mm) 3D	-0.096 ± 0.123	-0.104 ± 0.125	$[-0.380, 0.206]$	-0.024	-0.182	0.017
Peak pupil diameter (mm) 2D	0.314 ± 0.101	0.298 ± 0.084	$[0.109, 0.711]$	1.143	2.679	0.014
Peak pupil diameter (mm) 3D	0.416 ± 0.104	0.399 ± 0.071	$[0.213, 0.643]$	0.422	-0.323	0.014
Mean distance to figure (cm) 2D	88.599 ± 8.584	87.718 ± 6.232	$[68.196, 111.169]$	0.779	0.953	1.168
Mean distance to figure (cm) 3D	86.567 ± 10.210	84.507 ± 6.835	$[65.588, 127.626]$	1.458	3.865	1.389
Mean head movement to the sides (cm) 2D	4.942 ± 3.595	4.089 ± 3.399	$[0.227, 18.459]$	1.117	1.775	0.489
Mean head movement to the sides (cm) 3D	5.713 ± 3.438	5.113 ± 2.891	$[0.708, 18.177]$	1.061	1.404	0.468

Table 1. For each condition separately, we report mean ($\pm$ standard deviation), median ($\pm$ median absolute deviation), minimum and maximum, skewness, kurtosis, and standard error. All values are given on the participant level. Units are either seconds (s), number per second (n/s), a ratio between 0 and 1, or greater and smaller than 1 ($\lesseqgtr 1$), angle in degrees per second ($°/s$), millimeters (mm), centimeters (cm), or centimeters per second (cm/s).

Supplementary Table S2: Table of two-tailed, paired t-tests

**Feature**	**T**	**P**	**M diff**	95% **CI**	**Effect size**
Percentage solved correctly	-3.515	0.001	-0.05 ± 0.014	$[-0.078, -0.021]$	-0.478
Reaction time (s)	3.915	< 0.001	0.785 ± 0.2	$[0.383, 1.187]$	0.533
Mean fixation duration (s)	1.049	> 0.999	0.003 ± 0.003	$[-0.003, 0.008]$	0.143
Mean fixation rate (n/s)	-2.129	0.456	-0.062 ± 0.029	$[-0.121, -0.004]$	-0.29
Mean regressive fixation duration (s)	-7.324	< 0.001	-0.036 ± 0.005	$[-0.045, -0.026]$	-0.997
Equal fixation duration between figures (ratio)	-2.575	0.156	-0.026 ± 0.01	$[-0.047, -0.006]$	-0.35
Equal fixation duration within figures (ratio)	-24.423	< 0.001	-0.262 ± 0.011	$[-0.284, -0.241]$	-3.324
Strategy ratio ($\lesseqgtr 1$)	6.804	< 0.001	0.718 ± 0.106	$[0.507, 0.93]$	0.926
Mean saccade velocity ($^\circ/s$)	-6.672	< 0.001	-11.29 ± 1.692	$[-14.684, -7.896]$	-0.908
Mean saccade rate (n/s)	-3.911	< 0.001	-0.135 ± 0.035	$[-0.205, -0.066]$	-0.532
Mean pupil diameter (mm)	9.403	< 0.001	0.135 ± 0.014	$[0.106, 0.163]$	1.28
Peak pupil diameter (mm)	-9.302	< 0.001	-0.102 ± 0.011	$[-0.125, -0.08]$	-1.266
Mean distance to figure (cm)	2.985	0.048	2.033 ± 0.681	$[0.667, 3.398]$	0.406
Mean head movement to the sides	-3.582	0.012	-0.77 ± 0.215	$[-1.202, -0.339]$	-0.487

Table 2. Student's t-test comparing the 2D and 3D conditions ($df = 53$). We report the T statistics from the two-tailed, paired test. P values for all eye and head features were Bonferroni corrected. A positive mean difference value indicates a higher mean value in the 2D condition ($\pm standarderror$). The 95% confidence interval for the mean difference and Cohen's d effect size is reported. Units are either seconds (s), number per second (n/s), a ratio between 0 and 1, or greater and smaller than 1 ($\lesseqgtr 1$), angle in degrees per second ($^\circ/s$), millimeters (mm), centimeters (cm), or centimeters per second (cm/s).

Supplementary Table S3: Results on sex differences

Variable	Condition	Female ($M \pm SD$)	Male ($M \pm SD$)	F	P
Reaction time (s)	Total	6.173 ± 2.912	6.931 ± 3.907	0.815	0.420
	2D	6.551 ± 3.249	7.348 ± 4.089	0.794	0.431
	3D	5.796 ± 2.675	6.515 ± 3.949	0.799	0.428
Percentage solved correctly	Total	0.849 ± 0.091	0.869 ± 0.085	0.806	0.424
	2D	0.824 ± 0.11	0.845 ± 0.096	0.738	0.464
	3D	0.875 ± 0.095	0.893 ± 0.11	0.651	0.518

Table 3. This table states performance differences between sexes in the experiment. Mean and standard deviation for performance (reaction time in seconds and percentage solved correctly) for both sexes in the total experiment and each experimental condition. We report differences from two-tailed, unpaired t-tests comparing female and male participants.

Supplementary Table S4: Average mental rotation performance by condition and experiment order

Variable	Condition	Order	$M \pm SD$
Reaction time (s)	2D	first	7.639 ± 2.278
	3D	second	6.438 ± 3.066
	3D	first	5.588 ± 3.411
	2D	second	5.811 ± 3.000
Percentage solved correctly	2D	first	0.827 ± 0.083
	3D	second	0.915 ± 0.076
	3D	first	0.837 ± 0.114
	2D	second	0.839 ± 0.130

Table 4. This table states the performance between the experiment condition and the order in which the conditions were shown to the participants. Mean and standard deviation for performance (reaction time in seconds and percentage solved correctly) are reported. 23 participants (18 female and 8 male) started with 3D condition first and 31 (18 female and 13 male) with the 2D condition first.

Supplementary Table S5: Reaction time for rotation angles and condition

Condition	Rotation angle	Reaction time ($M \pm SD$)	Reaction time ($Med \pm MAD$)
2D	40°	5.994 ± 2.926	5.430 ± 1.329
	80°	7.226 ± 4.444	5.733 ± 1.974
	120°	7.176 ± 4.339	6.220 ± 1.481
	160°	7.397 ± 3.627	6.394 ± 1.632
3D	40°	5.384 ± 2.850	4.408 ± 1.065
	80°	5.918 ± 3.177	4.817 ± 1.123
	120°	6.453 ± 3.815	5.339 ± 1.477
	160°	6.700 ± 3.671	5.634 ± 1.745

Table 5. This table states the reaction time in seconds for all different rotation angles for each condition separately. Mean and standard deviation as well as median (Med) and median absolute deviation (MAD) are reported for all $n = 54$ participants.

Supplementary Table S6: Pearson's pairwise correlations between all eye movement features

	1	2	3	4	5	6	7	8	9	10	11
1 Mean fixation duration (s)	-	-	-	-	-	-	-	-	-	-	-
2 Mean fixation rate (n/s)	-0.16	-	-	-	-	-	-	-	-	-	-
3 Mean regressive fixation duration (s)	0.30	0.05	-	-	-	-	-	-	-	-	-
4 Equal fixation duration between figures (ratio)	-0.06	0.23	0.22	-	-	-	-	-	-	-	-
5 Equal fixation duration within figure (ratio)	-0.06	0.12	0.00	0.14	-	-	-	-	-	-	-
6 Strategy ratio ($\lesseqgtr 1$)	0.04	0.03	0.13	0.06	-0.06	-	-	-	-	-	-
7 Mean saccade velocity ($^\circ/s$)	-0.02	0.14	0.02	0.07	0.09	-0.14	-	-	-	-	-
8 Mean saccade rate (n/s)	-0.39	-0.01	-0.23	-0.12	-0.0	-0.31	-0.03	-	-	-	-
9 Mean pupil diameter (mm)	0.01	-0.08	0.0	0.01	-0.15	0.16	-0.06	-0.13	-	-	-
10 Peak pupil diameter (mm)	0.07	-0.01	0.07	0.02	0.12	-0.09	0.03	0.03	-0.23	-	-
11 Mean distance to figure (cm)	-0.03	0.1	-0.01	0.03	-0.04	0.03	-0.24	-0.02	-0.07	-0.1	-
12 Mean head movement to the sides (cm)	-0.01	0.02	0.0	0.06	0.09	0.01	0.07	-0.03	0.04	-0.04	0.05

Table 6. Pearson's pairwise correlations between all eye movement features. Units are either seconds (s), number per second (n/s), a ratio between 0 and 1, or greater and smaller than 1 ($\lesseqgtr 1$), angle in degrees per second ($^\circ/s$), millimeters (mm), centimeters (cm), or centimeters per second (cm/s).

Supplementary Table S7: Pearson's pairwise correlations between all eye movement features in the 2D condition

	1	2	3	4	5	6	7	8	9	10	11
1 Mean fixation duration (s)	-	-	-	-	-	-	-	-	-	-	-
2 Mean fixation rate (n/s)	-0.24	-	-	-	-	-	-	-	-	-	-
3 Mean regressive fixation duration (s)	0.25	0.04	-	-	-	-	-	-	-	-	-
4 Equal fixation duration between figures (ratio)	-0.08	0.26	0.29	-	-	-	-	-	-	-	-
5 Equal fixation duration within figure (ratio)	-0.04	0.07	-0.14	0.14	-	-	-	-	-	-	-
6 Strategy ratio ($\lesseqgtr 1$)	0.07	0.07	0.24	0.19	0.04	-	-	-	-	-	-
7 Mean saccade velocity ($^\circ/s$)	-0.06	0.1	-0.03	0.04	0.03	-0.1	-	-	-	-	-
8 Mean saccade rate (n/s)	-0.41	-0.04	-0.23	-0.11	-0.01	-0.35	-0.05	-	-	-	-
9 Mean pupil diameter (mm)	-0.0	0.02	0.05	0.03	0.01	0.09	0.03	-0.04	-	-	-
10 Peak pupil diameter (mm)	0.09	-0.08	0.02	-0.02	-0.04	-0.01	-0.04	-0.07	-0.01	-	-
11 Mean distance to figure (cm)	0.02	0.09	0.01	0.05	-0.0	0.02	-0.23	-0.02	-0.12	-0.1	-
12 Mean head movement to the sides (cm)	-0.01	0.03	-0.02	0.09	0.01	0.03	0.08	-0.04	0.03	-0.06	0.04

Table 7. Pearson's pairwise correlations between all eye movement featuresfor trials in the 2D condition. Units are either seconds (s), number per second (n/s), a ratio between 0 and 1, or greater and smaller than 1 ($\lesseqgtr 1$), angle in degrees per second ($^\circ/s$), millimeters (mm), centimeters (cm), or centimeters per second (cm/s).

Supplementary Table S8: Pearson's pairwise correlations between all eye movement features in the 3D condition

	1	2	3	4	5	6	7	8	9	10	11
1 Mean fixation duration (s)	-	-	-	-	-	-	-	-	-	-	-
2 Mean fixation rate (n/s)	-0.1	-	-	-	-	-	-	-	-	-	-
3 Mean regressive fixation duration (s)	0.36	0.04	-	-	-	-	-	-	-	-	-
4 Equal fixation duration between figures (ratio)	-0.03	0.2	0.12	-	-	-	-	-	-	-	-
5 Equal fixation duration within figure (ratio)	-0.07	0.15	-0.01	0.12	-	-	-	-	-	-	-
6 Strategy ratio ($\lesseqgtr 1$)	-0.03	-0.03	0.02	-0.25	0.08	-	-	-	-	-	-
7 Mean saccade velocity ($°/s$)	0.01	0.15	0.03	0.09	0.01	-0.2	-	-	-	-	-
8 Mean saccade rate (n/s)	-0.37	0.0	-0.27	-0.16	-0.1	-0.28	-0.05	-	-	-	-
9 Mean pupil diameter (mm)	0.0	-0.12	0.07	0.03	0.05	0.1	-0.02	-0.15	-	-	-
10 Peak pupil diameter (mm)	0.08	0.0	0.02	0.01	-0.04	-0.03	-0.0	0.05	-0.2	-	-
11 Mean distance to figure (cm)	-0.07	0.11	-0.01	0.03	0.02	-0.05	-0.23	-0.0	-0.12	-0.04	-
12 Mean head movement to the sides (cm)	-0.0	0.0	-0.0	0.02	0.08	0.04	0.04	-0.05	0.12	-0.1	0.08

Table 8. Pearson's pairwise correlations between all eye movement features for trials in the 3D condition. Units are either seconds (s), number per second (n/s), a ratio between 0 and 1, or greater and smaller than 1 ($\lesseqgtr 1$), angle in degrees per second ($°/s$), millimeters (mm), centimeters (cm), or centimeters per second (cm/s).

Supplementary Table S9: Differences between conditions and stimulus type

	Equal	Mirrored	Structural
Correct 2D	0.835 ± 0.372	0.837 ± 0.369	0.815 ± 0.389
Correct 3D	0.929 ± 0.258	0.829 ± 0.377	0.844 ± 0.363
RT (s) 2D	6.102 ± 4.674	7.424 ± 5.39	7.97 ± 6.077
RT (s) 3D	5.179 ± 3.801	6.427 ± 4.913	7.955 ± 6.712
Mean fixation duration (s) 2D	0.215 ± 0.046	0.225 ± 0.046	0.218 ± 0.046
Mean fixation duration (s) 3D	0.21 ± 0.047	0.22 ± 0.048	0.225 ± 0.047
Mean fixation rate (n/s) 2D	2.252 ± 0.507	2.205 ± 0.485	2.263 ± 0.473
Mean fixation rate (n/s) 3D	2.297 ± 0.613	2.298 ± 0.547	2.317 ± 0.484
Mean regressive fixation duration (s) 2D	0.133 ± 0.123	0.153 ± 0.13	0.145 ± 0.125
Mean regressive fixation duration (s) 3D	0.165 ± 0.118	0.186 ± 0.119	0.194 ± 0.109
Equal fixation duration between figures (ratio) 2D	0.687 ± 0.234	0.694 ± 0.216	0.718 ± 0.212
Equal fixation duration between figures (ratio) 3D	0.704 ± 0.226	0.715 ± 0.206	0.783 ± 0.171
Equal fixation duration within figure (ratio) 2D	0.162 ± 0.238	0.222 ± 0.252	0.196 ± 0.292
Equal fixation duration within figure (ratio) 3D	0.457 ± 0.25	0.424 ± 0.256	0.473 ± 0.236
Strategy ratio ($\lesseqgtr 1$) 2D	1.405 ± 2.085	1.476 ± 2.116	1.854 ± 2.358
Strategy ratio ($\lesseqgtr 1$) 3D	0.724 ± 0.699	0.787 ± 0.651	0.874 ± 0.669
Mean saccade velocity ($^\circ/s$) 2D	240.319 ± 34.66	239.823 ± 35.348	234.854 ± 34.467
Mean saccade velocity ($^\circ/s$) 3D	250.9 ± 37.714	247.237 ± 35.435	255.005 ± 36.977
Mean saccade rate (n/s) 2D	2.061 ± 0.691	1.948 ± 0.688	2.014 ± 0.68
Mean saccade rate (n/s) 3D	2.234 ± 0.684	2.126 ± 0.708	1.966 ± 0.638
Mean pupil diameter (mm) 2D	0.029 ± 0.147	0.036 ± 0.152	0.07 ± 0.166
Mean pupil diameter (mm) 3D	-0.095 ± 0.181	-0.111 ± 0.173	-0.07 ± 0.194
Pupil diameter amplitude (mm) 2D	0.314 ± 0.137	0.314 ± 0.135	0.314 ± 0.13
Pupil diameter amplitude (mm) 3D	0.419 ± 0.149	0.409 ± 0.155	0.419 ± 0.156
Mean distance to figure (cm) 2D	88.582 ± 8.548	88.664 ± 8.531	88.53 ± 8.547
Mean distance to figure (cm) 3D	86.523 ± 10.295	86.617 ± 10.293	86.597 ± 10.287
Mean head movement to the sides (cm) 2D	4.918 ± 3.595	5.007 ± 3.628	4.892 ± 3.608
Mean head movement to the sides (cm) 3D	5.817 ± 3.887	5.709 ± 3.883	5.428 ± 3.644

Table 9. Mean values for all features separated by the experiment condition and the three different stimulus types. Units are either seconds (s), number per second (n/s), a ratio between 0 and 1, or greater and smaller than 1 ($\lesseqgtr 1$), angle in degrees per second ($^\circ/s$), millimeters (mm), centimeters (cm), or centimeters per second (cm/s).

Supplementary Table S10: Interaction effects for conditions and stimulus type

	Interaction	Estimate	Z	P	95% CI
Percentage solved correctly	3D:(Mirrored-Equal)	-0.094 ± 0.028	-3.353	0.001	$[-0.149, -0.039]$
	3D:(Structural-Equal)	-0.060 ± 0.034	-1.746	0.081	$[-0.127, 0.007]$
RT (s)	3D:(Mirrored-Equal)	-0.046 ± 0.315	-0.145	0.885	$[-0.664, 0.573]$
	3D:(Structural-Equal)	0.877 ± 0.384	2.284	0.022	$[0.124, 1.630]$
Mean fixation duration (s)	3D:(Mirrored-Equal)	0.001 ± 0.003	0.238	1.0	$[-0.006, 0.007]$
	3D:(Structural-Equal)	0.011 ± 0.004	2.736	0.072	$[0.003, 0.018]$
Mean fixation rate (n/s)	3D:(Mirrored-Equal)	0.066 ± 0.037	1.800	0.864	$[-0.006, 0.138]$
	3D:(Structural-Equal)	0.014 ± 0.045	0.314	1.0	$[-0.074, 0.102]$
Mean regressive fixation duration (s)	3D:(Mirrored-Equal)	0.004 ± 0.009	0.395	1.0	$[-0.015, 0.022]$
	3D:(Structural-Equal)	0.017 ± 0.012	1.500	1.0	$[-0.005, 0.040]$
Equal fixation duration between figures (ratio)	3D:(Mirrored-Equal)	0.016 ± 0.016	0.993	1.0	$[-0.016, 0.049]$
	3D:(Structural-Equal)	0.051 ± 0.020	2.541	0.132	$[0.012, 0.090]$
Equal fixation duration within figure (ratio)	3D:(Mirrored-Equal)	-0.096 ± 0.020	-4.691	0.0	$[-0.136, -0.056]$
	3D:(Structural-Equal)	-0.021 ± 0.025	-0.865	1.0	$[-0.070, 0.027]$
Strategy ratio ($\lesseqgtr 1$)	3D:(Mirrored-Equal)	-0.015 ± 0.124	-0.123	1.0	$[-0.257, 0.227]$
	3D:(Structural-Equal)	-0.304 ± 0.150	-2.020	0.516	$[-0.599, -0.009]$
Mean saccade velocity ($^\circ/s$)	3D:(Mirrored-Equal)	-3.398 ± 2.441	-1.392	1.0	$[-8.181, 1.386]$
	3D:(Structural-Equal)	9.491 ± 2.971	3.194	0.012	$[3.668, 15.315]$
Mean saccade rate (n/s)	3D:(Mirrored-Equal)	0.009 ± 0.043	0.200	1.0	$[-0.076, 0.093]$
	3D:(Structural-Equal)	-0.213 ± 0.052	-4.083	0.0	$[-0.316, -0.111]$
Mean pupil diameter (mm)	3D:(Mirrored-Equal)	-0.023 ± 0.011	-1.996	0.552	$[-0.045, -0.000]$
	3D:(Structural-Equal)	-0.015 ± 0.014	-1.080	1.0	$[-0.042, 0.012]$
Pupil diameter amplitude (mm)	3D:(Mirrored-Equal)	-0.008 ± 0.009	-0.892	1.0	$[-0.026, 0.010]$
	3D:(Structural-Equal)	-0.001 ± 0.011	-0.101	1.0	$[-0.023, 0.021]$
Mean distance to figure (cm)	3D:(Mirrored-Equal)	0.008 ± 0.234	0.034	1.0	$[-0.450, 0.466]$
	3D:(Structural-Equal)	0.083 ± 0.284	0.293	1.0	$[-0.474, 0.641]$
Mean head movement to the sides (cm)	3D:(Mirrored-Equal)	-0.177 ± 0.128	-1.380	1.0	$[-0.427, 0.074]$
	3D:(Structural-Equal)	-0.332 ± 0.156	-2.134	0.396	$[-0.638, -0.027]$

Table 10. Interaction effects between the categorical variables experimental condition ($C2$ and $C3$) and stimulus type (SE, SM, SS) with 2D and equal as reference category. We report the estimates ± standard errors for interaction effects of multi-level regression models (β_4 and β_5, with participant's ID i as random intercept β_{0i}). One separate regression model was fitted for each feature as the dependent variable $Y(variable)$. The regression formula was: $\hat{Y}(variable)_{ij} = \beta_0 + \beta_{oi} + \beta_1 C3_{ij} + \beta_2 SM_{ij} + \beta_3 SS_{ij} + \beta_4 C3_{ij} SM_{ij} + \beta_5 C3_{ij} SS_{ij} + \varepsilon_{ij}$. P values for all eye and head features were Bonferroni corrected. Z statistic and the 95% confidence interval for the estimate are reported. Units are either seconds (s), number per second (n/s), a ratio between 0 and 1, or greater and smaller than 1 ($\lesseqgtr 1$), angle in degrees per second ($^\circ/s$), millimeters (mm), centimeters (cm), or centimeters per second (cm/s).
